# Computational identification of specific genes for glioblastoma stem-like cells identity

**DOI:** 10.1038/s41598-018-26081-5

**Published:** 2018-05-17

**Authors:** Giulia Fiscon, Federica Conte, Valerio Licursi, Sergio Nasi, Paola Paci

**Affiliations:** 1 Institute for Systems Analysis and Computer Science “Antonio Ruberti”, National Research Council, Rome, Italy; 2 SysBio Centre of Systems Biology, Rome, Italy; 3 Department of Biology and Biotecnology - Charles Darwin, “Sapienza” University of Rome, Rome, Italy; 4 Institute of Molecular Biology and Pathology (IBPM), National Research Council (CNR), Rome, Italy

## Abstract

Glioblastoma, the most malignant brain cancer, contains self-renewing, stem-like cells that sustain tumor growth and therapeutic resistance. Identifying genes promoting stem-like cell differentiation might unveil targets for novel treatments. To detect them, here we apply SWIM – a software able to unveil genes (named switch genes) involved in drastic changes of cell phenotype – to public datasets of gene expression profiles from human glioblastoma cells. By analyzing matched pairs of stem-like and differentiated glioblastoma cells, SWIM identified 336 switch genes, potentially involved in the transition from stem-like to differentiated state. A subset of them was significantly related to focal adhesion and extracellular matrix and strongly down-regulated in stem-like cells, suggesting that they may promote differentiation and restrain tumor growth. Their expression in differentiated cells strongly correlated with the down-regulation of transcription factors like OLIG2, POU3F2, SALL2, SOX2, capable of reprogramming differentiated glioblastoma cells into stem-like cells. These findings were corroborated by the analysis of expression profiles from glioblastoma stem-like cell lines, the corresponding primary tumors, and conventional glioma cell lines. Switch genes represent a distinguishing feature of stem-like cells and we are persuaded that they may reveal novel potential therapeutic targets worthy of further investigation.

## Introduction

Glioblastoma multiforme (GBM) is the most aggressive and frequent brain tumor, with a median survival time of only 12–15 months from diagnosis^1–3^. It accounts for 15% of all primary brain tumors, 46% of primary malignant brain tumors and around 60–75% of astrocytomas. The frequency of GBM–which affects more men than women–increases with age and the tumor becomes more common over age 45 ^3,4^. GBM shows a high infiltration into the brain parenchyma, making standard therapies (*e.g*. surgical resection, followed by radiotherapy and chemotherapy with temozolomide) unable to effectively arrest tumor development and progression^5,6^. The GBM mortality rate is extremely high when compared to other cancers such as breast and lung cancer^4^, with the 5-years survival rate achieved for only 5% of patients^7–10^. While GBM remains incurable, current research and clinical trials have contributed to a better understanding of the disease progression and to small improvements in patient outcomes. In particular, several studies identified a subpopulation of GBM cells with radio/chemotherapy-resistant properties that have a role in driving tumor initiation, progression, resistance to treatment, and relapse^11–18^. Due to their abilities of self-renewal, proliferation, and differentiation into multiple lineages, these cells are named glioblastoma stem-like cells (GSCs) or tumor-propagating cells (TPCs)^19^, and are held responsible for carcinogenesis. Stem-like cells are not unique to GBM, but they are present in several other cancers, such as breast, colon, prostate, pancreatic cancer, and melanoma^11,14,20–24^. The failure to remove these cancer stem-like cells is believed to be one of the main reasons behind the ineffectiveness of current therapies in treating glioblastoma and other cancers^17^. Triggering differentiation of cancer stem-like cells may represent a therapeutic opportunity for glioblastoma. Therefore, it is important to better elucidate the factors that govern their fate.

Increasing evidence suggests that cell fate decisions in a variety of cell types can be overridden by the artificial expression of a small set of transcription factors (TFs). A recent study^13^ identified 19 neurodevelopmental TFs that are selectively expressed in GSCs to maintain their stem-like phenotype and prevent differentiation. A subset of only four of them–SOX2, OLIG2, POU3F2, and SALL2–was sufficient to fully reprogram differentiated cells into glioblastoma stem-like cells^13,25–27^. In particular, SOX2 is a controller of stem cell pluripotency that may also function as a switch in neuronal development and is strongly associated with the maintenance of the undifferentiated state of cancer stem cells in several tissues^28–30^. OLIG2 is a promoter of oligodendrocyte differentiation and a negative regulator of neuronal differentiation, which has a pivotal role in GBM by supporting proliferation and self-renewal of GSCs^31–33^. Finally, POU3F2 plays a key role in neuronal differentiation and SALL2 is widely expressed in brain and may have a role in promoting neuronal development^13^.

Recently, we developed the software SWIM (SWItchMiner)^34^, which is able to unveil from genome-wide expression data a pool of peculiar genes–called “switch genes”–that are expected to be critically associated with drastic changes in the phenotype of cells or tissues. SWIM gave promising results in the study of the grapevine developmental shift from the immature to the mature growth phase^35^, as well as in a multi-cancer analysis^34^.

Motivated by the key role of stem-like cells in glioblastoma propagation and therapeutic resistance, here we applied SWIM to computationally identify genes involved in the transition from a stem-like to a differentiated phenotype of glioblastoma cells. To this end, we investigated gene expression profiles from two datasets, publicly available on the Gene Expression Omnibus (GEO) repository^36^ (i): RNA sequencing data obtained from stem-like tumor-propagating cells and differentiated glioblastoma cells (DGCs)^13^ (ii); Affymetrix HG-U133 Plus 2.0 microrarrays expression data from glioblastoma stem-like cell lines, the corresponding primary tumors, and conventional glioma cell lines^37^. The switch genes that we have identified in this work could have the potential to improve our knowledge of the cellular functions that are crucial for glioblastoma, such as the control of cancer stem-like cells differentiation and invasion.

## Results and Discussion

### Glioblastoma correlation network

**Figure 1:**
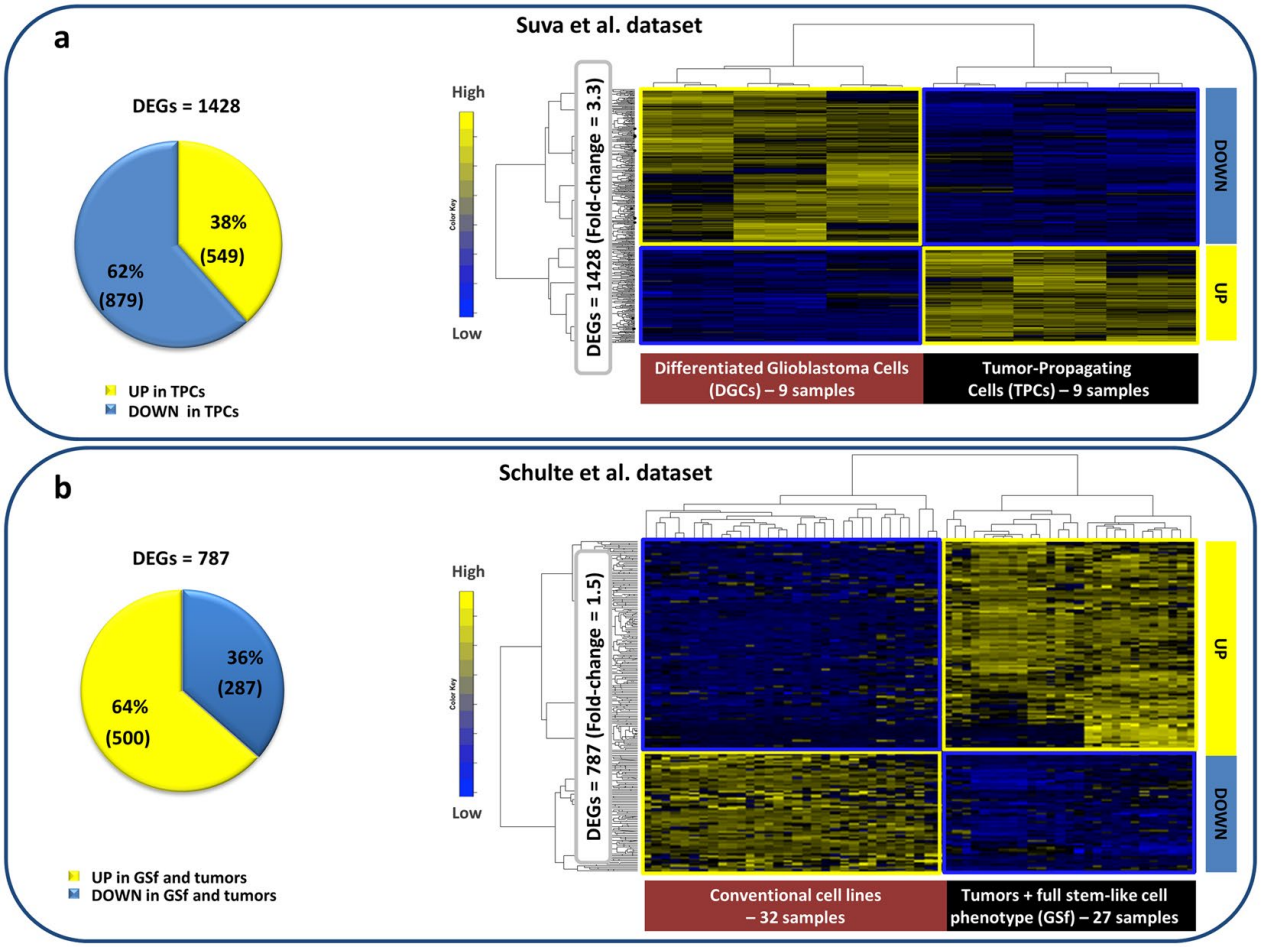
Differential gene expression analysis of glioblastoma cells. The pie charts in both panels represent the percentages of differentially expressed genes (DEGs) of Suva *et al*. dataset (**a**) that are up-/down-regulated in tumor-propagating cells (TPCs) in comparison to the differentiated glioblastoma cells (DGCs), and of Schulte *et al*. dataset (**b**) that are up-/down-regulated in glioblastoma full stem-like phenotype (GSf) cell lines and corresponding primary tumors in comparison to conventional cell lines. In both panels the figure show the dendrogram and the heat map of DEGs in Suva *et al*. dataset (**b**) and in Schulte *et al*. dataset. (**d**) The DEGs expression profiles are clustered according to genes (rows) and cells (columns) in the glioblastoma data matrices by using Pearson correlation distance as metrics. Heat map colors represent different expression levels increasing from blue to yellow.

In order to identify genes that may control the differentiation of stem-like tumor propagating cells into differentiated glioblastoma cells, we analyzed by SWIM GSE54792 dataset, from Suva and coworkers^13^. The dataset contains the global expression profiles obtained by RNA sequencing of matched pairs of cells derived from three different human tumors and grown either as stem-like tumour propagating cells or as differentiated glioblastoma cells. In the original study^13^, the authors identified four TPC-specific transcription factors–SOX2, OLIG2, POU3F2, SALL2–that play a key role in the TPCs to DGCs transition and focused on the expression of their target genes in the two alternative cellular states. Differently from their study, our analysis was purely computational and the transcriptome was investigated without *a-priori* information, searching for all possible master regulators of the differentiation of TPCs into DGCs. By performing a hierarchical clustering of the gene expression profiles, we found a clear-cut separation between the two cellular states (Supplementary Figure 1), indicating that the transition from stemness to differentiation is accompanied by a radical shift in the gene expression pattern. We then made use of SWIM in order to shed light on the driver genes of this fundamental shift. SWIM identified 1428 differentially expressed genes (DEGs) between TPCs and DGCs (Fig. 1a, Supplementary Table S1). The majority of them (62% corresponding to 879 genes) was down-regulated in TPCs vs DGCs (Fig. 1a), whereas the remaining minority (38% corresponding to 549 genes) was up-regulated and significantly enriched in the 19 neurodevelopmental TFs that are specifically active in TPCs^13,38^.

To clarify the roles of the differentially expressed genes, we investigated their functional annotations and over-represented pathways by means of FIDEA^39^ and GSEA^40^ tools, at an adjusted p-value threshold of 0.05. The KEGG pathways most significantly over-represented among the transcripts down-regulated in TPCs were “ECM-receptor interaction” and “focal adhesion” (Supplementary Table S2). The extracellular matrix (ECM) is commonly deregulated and becomes disorganized in cancer. ECM anomalies may affect cancer progression and directly promote tumor invasiveness^41^. Thus, understanding how ECM deregulation influences glioblastoma progression may help to develop new therapeutic interventions by targeting genes directly involved in ECM composition in brain. All these findings indicate that the activation of genes related to cell adhesion is likely to have a major role in the transcriptional reprogramming associated with the transition of glioblastoma cells from stemness to differentiation.

To further assess the validity of the SWIM analysis in identifying the distinguishing features of glioblastoma stem-like cells, we investigated a second dataset, GSE23806^37^, which contains the expression profiles of 15 glioblastoma full stem-like phenotype (GSf) cell lines, 12 corresponding primary tumors, and 32 conventional glioblastoma cell lines. SWIM extracted 787 genes that were differentially expressed between the conventional cell lines and the set of GSf and primary tumors (Fig. 1b, Supplementary Table S1). Notably, the list of down-regulated genes in GSf cells and primary tumors (36% of all DEGs) was enriched in “ECM-receptor interaction“ and “focal adhesion” KEGG cell communicationpathways (Supplementary Table S2), mirroring the results obtained by the analysis of the DEGs in the previous dataset^13^. This strengthens the idea that repression of cell adhesion and cell communication pathways is a key, distinguishing feature of glioblastoma stem-like cells.

From DEG expression profiles in the dataset by Suva *et al*.^13^, SWIM generated a correlation network using as a distance metric the Pearson correlation coefficient between any given pair of transcripts. Altogether, the co-expression network comprised 1428 nodes and 275954 edges (Supplementary Table S3). Nodes in the correlation network represent RNA transcripts and a link (edge) is drawn between two nodes if the absolute value of the Pearson correlation coefficient between their expression profiles exceeds a given threshold. The topological properties of the correlation network were investigated by classifying each hub (*i.e*. nodes with degree at least equal to 5 ^42^) as date, party, or fight-club on the basis of the Average Pearson Correlation Coefficient (APCC) between its expression profile and that of its nearest neighbors (Supplementary Figure 2)^34,35^. SWIM identified 1427 hubs (Supplementary Table S4): 136 party hubs, 849 date hubs, and 442 fight-club hubs (see the original paper^34^ for an explanation of this classification).

**Figure 2:**
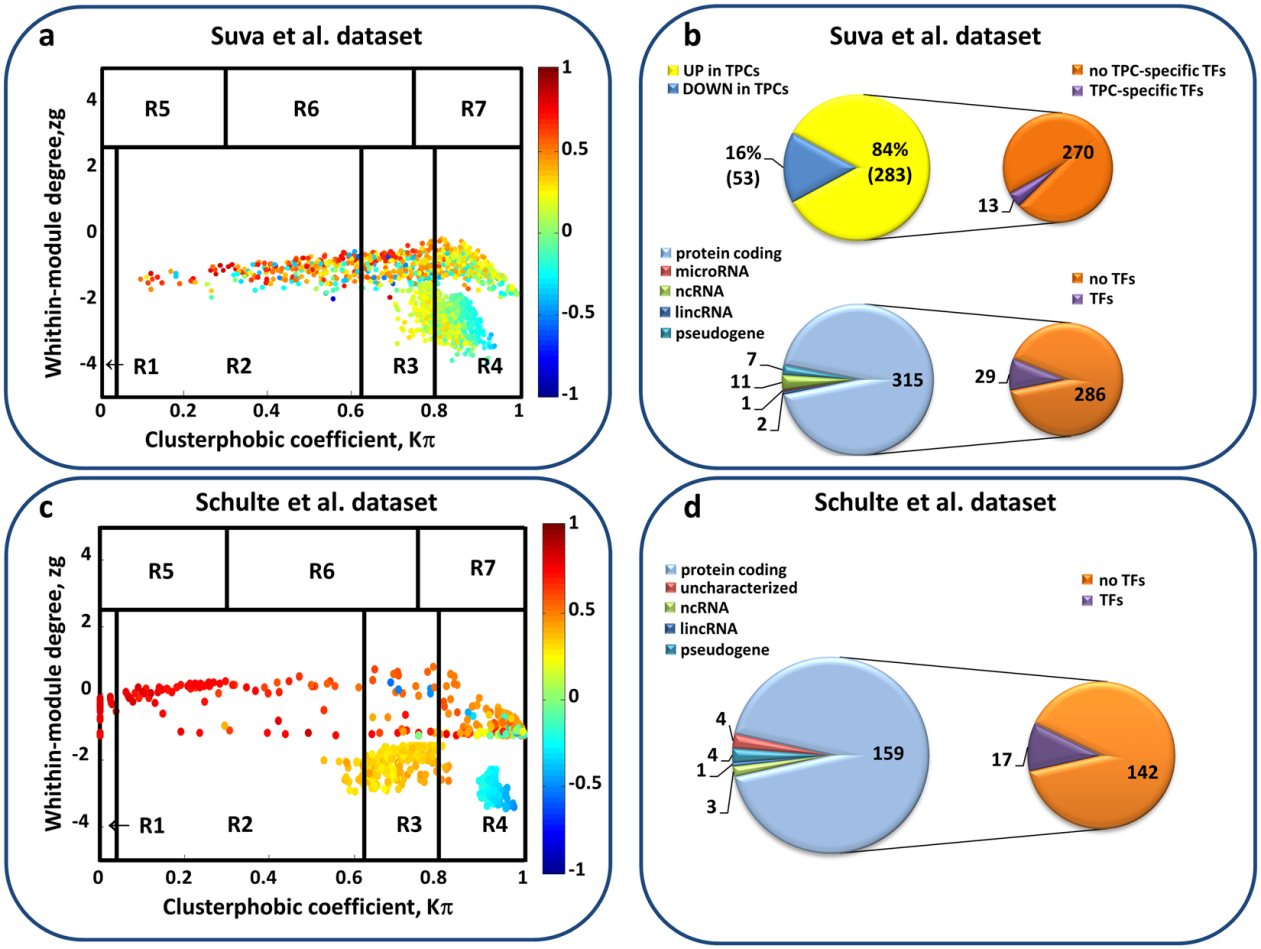
Identification and characterization of switch genes. (**a**–**c**) Heat cartography maps of nodes of the glioblastoma correlation networks from Suva *et al*. dataset (**a**) and from Schulte *et al*. dataset (**c**). Dots correspond to nodes in the glioblastoma correlation networks and are distributed across seven regions (R1 to R7) according to their clusterphobic coefficient *K*_*π*_ (x-axis), which is a measure of the “fear” of each node of being confined in its own cluster, and according to their within-module degree *Z*_*g*_ (y-axis). Each node is colored according to the value of the Average Pearson Correlation Coefficient (APCC) between its expression profile and that of its nearest neighbors in the network. (**b**-top) The larger pie charts illustrate the percentages of switch genes of Suva *et al*. dataset that are up-/down-regulated in the transition from tumor-propagating cells (TPCs) to differentiated glioblastoma cells (DGCs). The smaller pie charts highlight the number of TPC-specific transcription factors encompassed among the down-regulated switch genes. (**b**-bottom) The larger pie charts illustrate the switch genes classification of Suva *et al*. dataset according with their molecular type. The smaller pie charts highlight the number of transcription factors included among the protein coding switch genes. (**d**) The larger pie chart represents the classification of switch genes of Schulte *et al*. dataset according with their molecular type. The smaller pie charts highlight the number of transcription factors encompassed among the protein coding switch genes.

In order to assign a role to each node in the correlation network, SWIM firstly searched for clusters (or communities) using the k-means algorithm^43^ (Supplementary Figure 3) and then drew the heat cartography map^34,35^ by evaluating two coordinates related to their intra- and inter-modular connections (Fig. 2a): the clusterphobic coefficient *K*_*π*_ (which measures the links of each node to nodes outside its own cluster) and the within-module degree *Z*_*g*_ (which measures how “well-connected” each node is within its own cluster). Nodes having much more external than internal links present high *K*_*π*_ values, whereas high *Z*_*g*_ values denote nodes that are hubs within their community (local hubs). The cartography contains seven regions corresponding to the seven different topological features of the network nodes. In the heat cartography map of Fig. 2a, each node (*i.e*. hubs and non-hubs) is colored according to its APCC value. Switch genes are defined as the subset of fight-club nodes–which are colored in light blue or blue–present in the R4 region of the map^34^. Finally, switch genes present the following features:

1. they mainly interact outside their own cluster (high values of *K*_*π*_).
2. they are not local hubs (low values of *Z*_*g*_).
3. they are mainly anti-correlated with their interaction partners (negative APCC).

**Figure 3:**
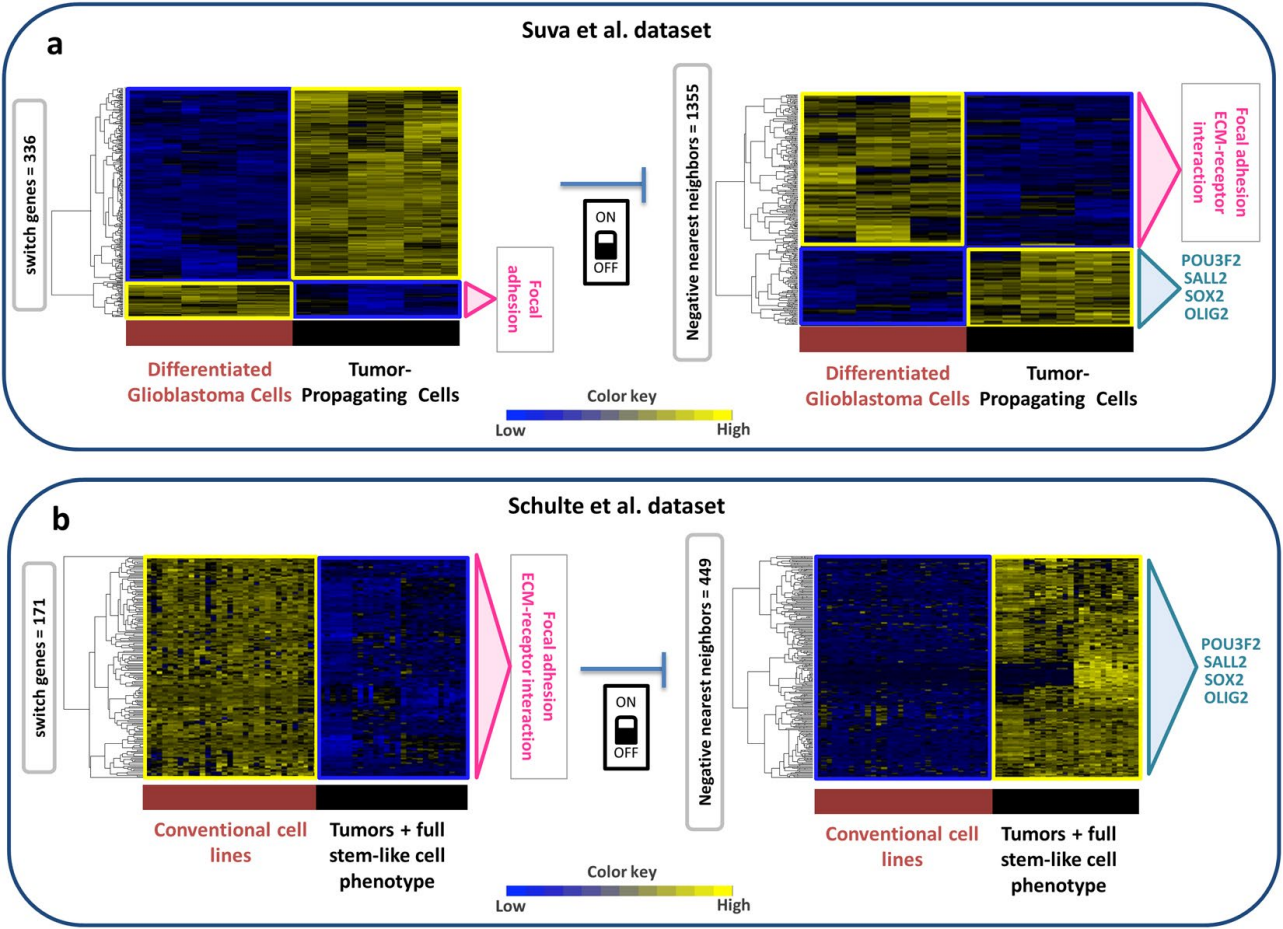
Switch genes and their negative nearest neighbors in the GBM networks. (**a**,**b**) Dendrogram and heat map of switch genes (left) and of their negative nearest neighbors (right) for Suva *et al*. dataset (**a**) and for Schulte *et al*. dataset (**b**) with their corresponding enriched pathways (Supplementary Table S12). The expression profiles of the switch genes and their negative nearest neighbors are clustered according to genes (rows) and cells (columns) in the glioblastoma data matrices, using Pearson correlation distance as metrics. Heat map colors represent different expression levels increasing from blue to yellow.

SWIM identified 336 switch genes out of 442 fight-club hubs (76%) in the Suva *et al*. glioblastoma dataset^13^, encompassing 20 long non-coding RNAs (*i.e*. 7 pseudogenes, 2 lincRNAs, and 11 antisense ncRNA), 1 microRNA, and 315 protein coding RNAs (Fig. 2b, Supplementary Tables S5, S6). Among the protein coding switch genes, 29 encoded transcription factors: all of them were up-regulated in TPCs except FOSL1, SMAD6, and CITED2 (Fig. 2b, Supplementary Table S6). Interestingly, SMAD6 is known to promote neuronal differentiation by inhibiting the Wnt pathway^44^ and FOSL1 is a regulator of cell adhesion and migration. Many of the up-regulated TFs may function as oncogenes by enhancing developmental programs required for tumorigenesis^45^. Accordingly, among the 26 TF switch genes up-regulated in TPCs, we found 13 of the 19 TPC-specific TFs (Fig. 2b, Supplementary Table S7), which direct the epigenetic state of TPCs^13^. They included SOX2, OLIG2, and POU3F2, which represent three out of the four core TFs, whose induction is sufficient to reprogram DGCs into TPCs^13^. Finally, the 336 switch genes were found to be negatively correlated to 1355 DEGs (Fig. 3a) and positively correlated to 1189 DEGs.

The GBM correlation network built from the Schulte *et al*. dataset^37^ comprises 732 nodes and 75209 edges (Supplementary Table S3). From this network, SWIM extracted 171 switch genes (Fig. 2c), which were found to be negatively correlated to 449 DEGs (Fig. 3b) and positively correlated to 224 DEGs. All switch genes were down-regulated in GSf cell lines and in the corresponding primary tumors, compared to the conventional glioblastoma cell lines (Supplementary Table S5). Switch genes include 159 protein-coding, 8 long non-coding (*i.e*. 4 pseudogenes, 3 antisense genes and 1 lincRNA), and 4 uncharacterised transcripts (Fig. 2d, Supplementary Table S6). Among the protein-coding switch genes, we found 17 encoded transcription factors.

Notably, the switch genes identified by analyzing both datasets^13,37^ were totally absent in similar heat cartography maps drawn with the nodes of randomized GBM gene expression networks obtained by shuffling the edges but preserving the degree of each node (Supplementary Figure 4).

### Switch genes characterization

To elucidate the possible role of switch genes in supporting the key features of glioblastoma cells, they were characterized on the basis of their expression and functional role. For what concerns the Suva *et al*. dataset^13^, we found that 84% of the switch genes were up-regulated in TPCs, suggesting that they may be involved in the maintenance of the stem-like features, whereas the remaining 16% were up-regulated in DGCs, indicating that they may support cell differentiation (Fig. 2b, Supplementary Table S5, Fig. 3a). The functional annotations showed that switch genes cover a variety of functions (Supplementary Table S8). The “focal adhesion” pathway was significantly over-represented (adjusted p-value < 0.05) among the switch genes up-regulated in DGCs (Fig. 3a and Supplementary Table S9). Their high expression in DGCs is strongly correlated with the inhibition of the key TFs like OLIG2, POU3F2, SALL2, and SOX2^13^ (Fig. 3a). These findings suggest that on one hand their activation may promote differentiation and restrain tumor growth, on the other hand their repression may contribute to tumor invasiveness. Indeed, the invasive process of cancer cells requires the loss of cell-cell adhesion, which allows malignant cells to dissociate from the primary tumor mass, and changes in the interaction with extracellular matrix, which enable the cells to invade the surrounding environment. This involves the secretion of substances able to degrade the extracellular matrix and the inhibition of proteins involved in the control of motility and migration^46^. Strikingly, all these considerations have been confirmed by the results obtained with the Schulte *et al*. dataset^37^, where the switch genes down-regulated in stem-like cells were found to be enriched in “ECM-receptor interaction” and “focal adhesion” pathways and highly anti-correlated with the four core TFs OLIG2, POU3F2, SALL2, SOX2 (Fig. 3b and Supplementary Table S9).

Taken together these results strongly supports our hypothesis of a potential involvement of switch genes related to cell communication pathways in controlling the stem-like phenotype of GBM cells by direct repression of the four core TFs. This would cause the induction of differentiation of cancer stem cells and severely halt cancer growth and invasion.

Searching for switch genes shared by both datasets, the FOS like transcription factor FOSL1 appears as the brightest star since i) it was down-regulated in stem-like cells and highly negatively correlated with the four core, TPC-specific TFs; ii) a consensus FOSL1 binding motif is present in the regulatory regions of all four core TFs, according to Pscan analysis^47^; and iii) it was shown to be involved in focal adhesion and migration in an *in vitro* mouse model of embryonic development^48^. In fact, FOSL1 was shown to function as a modulator of the level of key molecules on endothelial cell surface. It may act as activator or repressor, depending on the gene-context, and controls the delicate equilibrium between adhesion and migration^48^.

**Figure 4:**
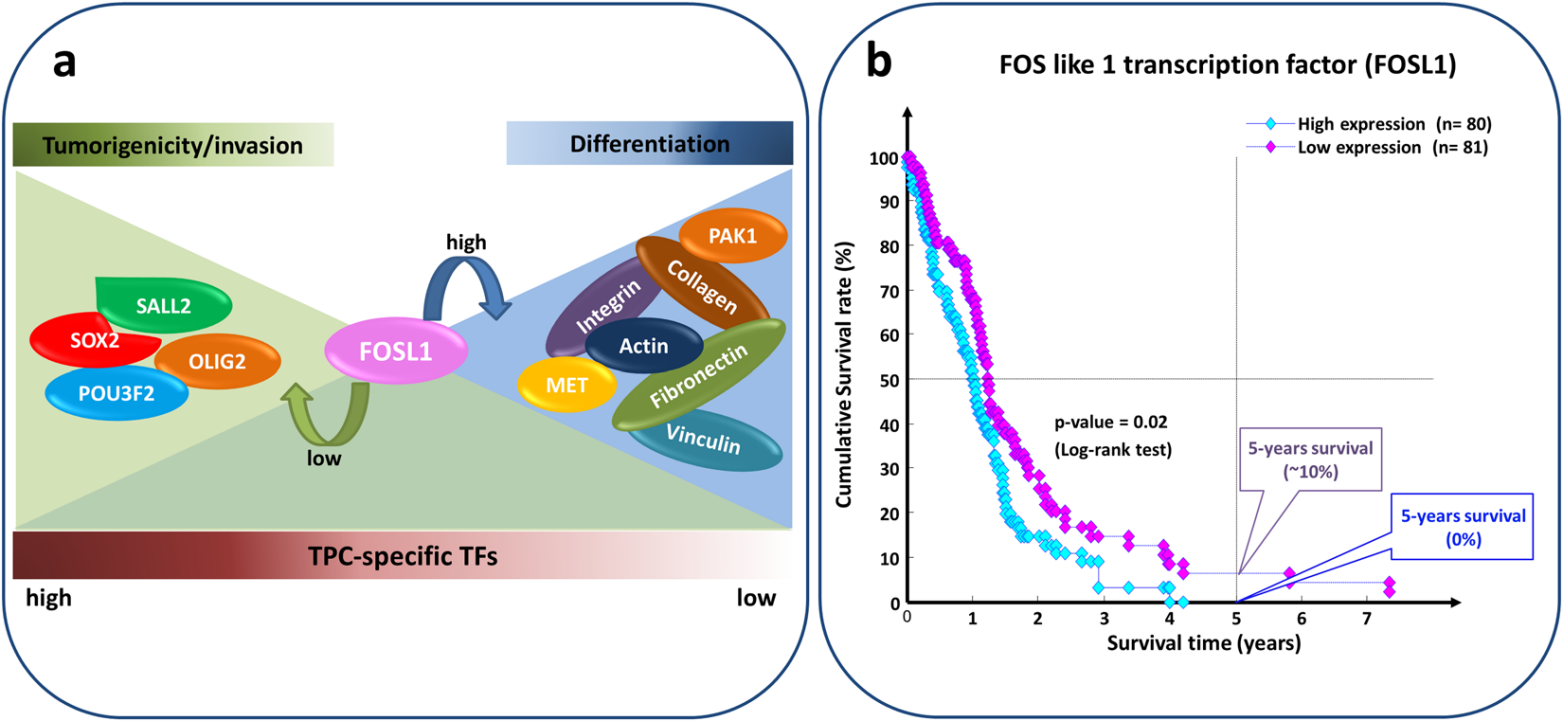
Potential role of FOSL1 in glioblastoma. (**a**) FOSL1 appears to be up-regulated in DGCs of Suva *et al*. dataset (*i.e*. DGCs) and in conventional glioma cell lines of Schulte *et al*. dataset. It is positively correlated with genes encoding proteins linked to focal adhesion complex and ECM receptor interaction, such as integrins, collagen, MET, PAK, and signaling proteins. Conversely, it is negatively regulated with TPC-specific TFs including the core set of OLIG2, POU3F2, SALL2, SOX2. Thus, it could be considered as putative controller of stem-like cell differentiation process by repressing the core set of neurodevelopmental TFs and by modulating the equilibrium between cell adhesion and migration (**b**) FOSL1 stands out in the top-ranked list of switch genes for both Suva *et al*. and Schulte *et al*. dataset as unfavorable prognostic marker. The sorted list was obtained by using Kaplan-Meier survival analysis based on the gene expression and clinical data of 161 GBM patients provided by TCGA. Patients were split according to a median separation into two groups (*i.e*. low- and high- groups refer to patients with expression levels lower than or greater than the 50^*th*^ percentile of the distribution of FOSL1 expression values).

Whilst on the one hand the expression of FOSL1 can promote the differentiation process by repressing TPC-specific transcription factors, on the other hand we found FOSL1 positively correlated with genes encoding proteins crucial for cell-matrix adhesion and cell motility, such as actin, collagen, fribonectin, and several integrins (Fig. 4a). In particular, integrins are transmembrane receptors that recruit ECM proteins like fibronectin and collagen, and transmit signals to the actin cytoskeleton through multiple bridging proteins such as vinculin and actinin. The engagement of the integrins with ECM proteins leads to formation of a focal adhesion complex that mechanically links intra-cellular actin bundles with extracellular environment. Changes in expression profiles of genes involved in this integrin-mediated process can influence cell adhesion dynamics and migration, and consequently increase cancer invasive behavior^41,49^.

### Prognostic role of switch genes in GBM

**Figure 5:**
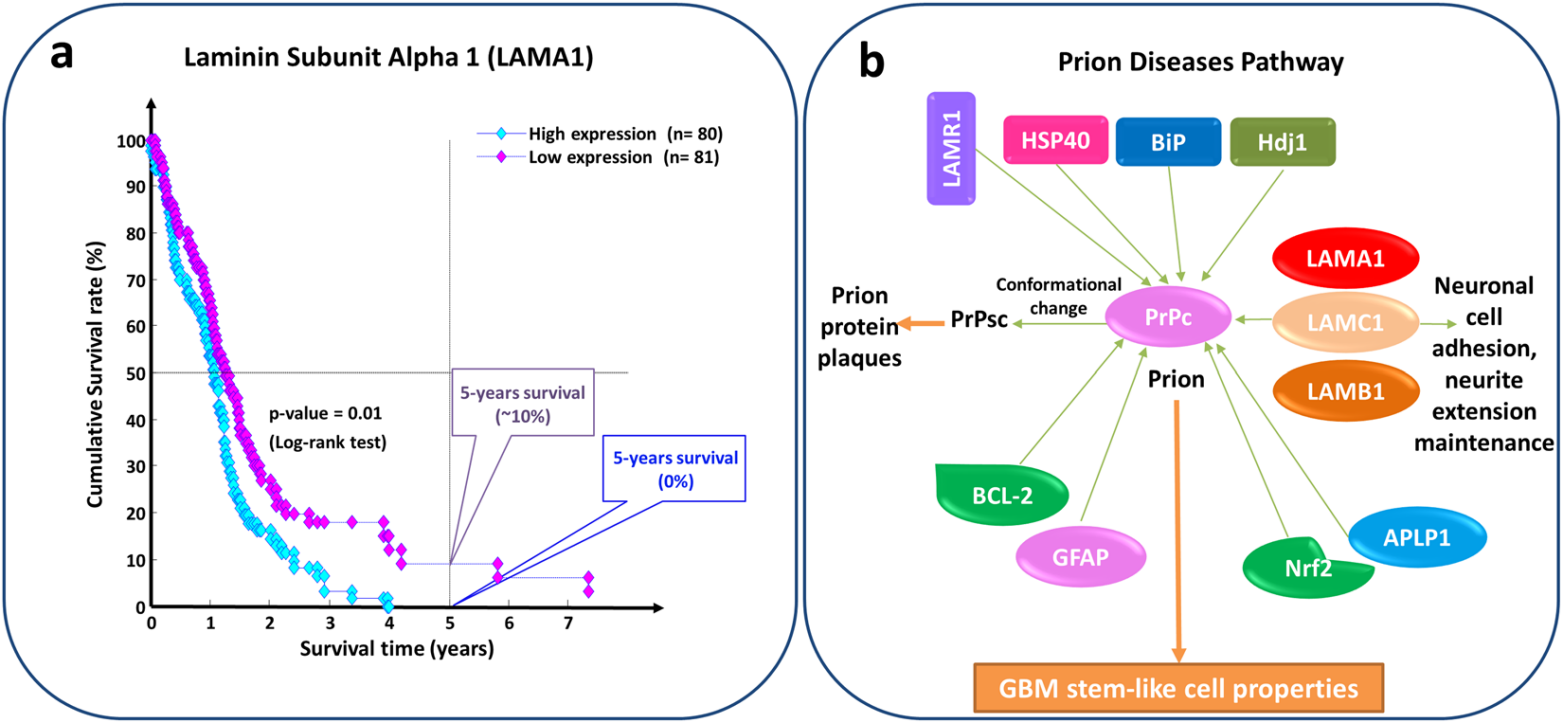
Potential role of LAMA1 in glioblastoma. (**a**) LAMA1 was amongst the top five glioblastoma switch genes of Suva et. al dataset with a statistically significant prognostic value. The sorted list was obtained by using Kaplan-Meier survival analysis based on the gene expression and clinical data of 161 GBM patients provided by TCGA. Patients were split according to a median separation into two groups (*i.e*. low- and high- groups refer to patients with expression levels lower than or greater than the ${50}^{th}$ percentile of the distribution of LAMA1 expression values). (**b**) Sketch of prion diseases pathway in which LAMA1 (highlighted in red) is involved.

To assess the possible relevance of switch genes as prognostic markers for GBM, we retrieved from the TCGA repository the gene expression and survival data of 161 GBM patients^50,51^. To determine whether a switch gene expression level might be significantly associated to lower or higher survival in the patient set, we performed a Kaplan-Meier analysis for each switch gene identified by SWIM in both datasets. To evaluate statistical significance, a p-value was assigned to each one as described in Materials and Methods, and the switch genes were sorted by increasing p-value in order to identify the best at separating the two prognosis groups (Supplementary Table S10). Once again FOSL1 drew our attention since it emerges in the top-ranked list of switch genes in both datasets: its high expression correlates with an unfavorable outcome (Fig. 4b). Among the top ten, we found also several genes known to be involved in glioblastoma, including the carbonic anhydrase CA14, the semaphorin SEMA6A, the metabolic enzyme GCSH, the ubiquitin ligase RNF135, and the laminin subunit LAMA1^52–56^. All these genes are candidate markers of GBM prognosis and sensitivity to therapy and might also represent therapeutic targets. We found particularly interesting LAMA1, which is an extracellular matrix glycoprotein. A lower expression of LAMA1 is associated with improved survival in the GBM patient dataset (Fig. 5a), whereas LAMA1 over-expression was found to correlate with increased tumor growth in primary glioblastomas^57^. Notably, LAMA1 may have a role in the pathway of prion diseases (Fig. 5b)–a group of fatal, transmissible spongiform encephalopathies^58,59^–by stimulate the prion protein activity. Interestingly, a recent study showed that the prion protein is able to enhance the stemness properties of GBM stem-like cells^60^, which is in agreement with our findings of LAMA1 up-regulation in TPCs.

### microRNAs targeting switch genes

Regarding the post-transcriptional control of switch genes from Suva *et al*. dataset, we focused on microRNAs (miRNAs). We searched by TargetScan^61^ the miRNAs that might bind to 3′UTRs of switch genes and by miRTarBase^62^ the miRNAs whose interactions with switch genes resulted experimentally validated. We first performed a miRNA target enrichment analysis of the list of switch genes by using TargetScan predictions and we found that this list was significantly enriched in targets of miRNAs implicated in regulating stem cell self-renewal and differentiation^12,63–69^ (Supplementary Figure 5 and Supplementary Table S11). Such miRNAs included miR-218, miR-29b, all members of miR-200 family^63,64,67,70–74^, and miR-21^75–78^. In particular, miR-218, the miR-200 family, and miR-29b were shown to inhibit glioblastoma invasion, migration, proliferation, and stemness through different targets^63,64,67,72–74^. In contrast, miR-21 - which is up-regulated in various types of cancer- has been associated with high proliferation, low apoptosis, and high migratory and invasive abilities in glioblastoma cells^75,76,78–81^. Additionally, miR-21 expression is positively correlated with the glioma grade and inversely correlated with survival of patients with glioma^77,82^. Finally, we performed a miRNA target enrichment analysis of the list of switch genes by using miRTarBase^62^ and we found miR-335 and miR-215 as the most enriched miRNAs with the highest number of targets among switch genes (Supplementary Table S11). Interestingly, miR-335 is capable of inducing glioma cell differentiation by activating cAMP/protein kinase A (PKA) pathway^83^, while the induction of miR-215 serves for maintaining glioblastoma stem-like cells^84^.

## Conclusions

Our findings show that the switch genes have the potential to improve our knowledge of the cellular events that are crucial for glioblastoma development. They may also provide important clues that might stimulate research activities to identify the drivers of this terrible disease and so support the rational planning of disease prevention or treatment. It’s worth to stress that this analysis should be regarded as a starting point and switch genes as putative interesting genes, whose importance should be experimentally tested. However, since the number of switch genes–even if much lower that the whole transcriptome–is still a high number, further investigations by experts and data integration are needed to better assess their functional and clinical relevance.

## Materials and Methods

### Datasets

#### Suva *et al*

The first GBM dataset analyzed for the present study is available through the GEO public repository at accession number GSE54792 published on Apr 11, 2014^13^. Data include genome-wide expression profiles obtained by RNA sequencing (Illumina HiSeq. 2000–2500) of matched pairs of GBM cultures derived from three different human tumors either as stem-like tumor-propagating cells (TPCs) grown in serum-free medium, spherogenic culture, or as differentiated glioblastoma cells (DGCs) grown as adherent monolayers in serum^13^.

#### Schulte *et al*

The second GBM dataset analyzed for the present study is available through GEO public repository under accession number GSE23806 published on Feb 12, 2011^37^. Data include expression profiles–obtained by Affymetrix Human Genome U133 Plus 2.0 Array–of 32 conventional glioma cell lines, 12 glioblastoma stem-like (GS) cell lines, among which 7 clonal sublines derived from two GS lines, 12 original tumors, from which GS-lines were derived, and 4 monolayer cultures established from the same tumors as GS-lines using standard serum conditions. The authors showed that only one subgroup of GS cell lines, called full stem-like phenotype (GSf), fulfilled all criteria for glioma stem cells and mirrored the transcriptome of human glioblastomas more closely than other cell lines. For this reason, in our analysis we compared the expression profiles of 23531 genes in 15 GSf cell lines and 12 corresponding primary tumors with respect to 32 conventional glioblastoma cell lines.

### SWIM software

SWIM^34^ is a software with a user-friendly Graphical User Interphase (GUI), developed in MATLAB and downloadable from Supplementary information of ref.^34^ where a detailed description can also be found. Briefly, SWIM computes the differentially expressed genes (adjusted p-value threshold of 0.05, fold-change threshold of 3.3), builds a correlation network of gene expression data (Person correlation threshold of 0.8) and identifies communities in the network by means of the k-means clustering algorithm, employing SSE (Sum of Squared Errors) values to determine the appropriate number of clusters (*k* = 3). It then creates a heat cartography map of the nodes according to their topological properties; it then extracts a select set of genes, named switch genes, which are expected to mark the shift from one condition to another in a complex biological network.

### microRNA target enrichment analysis

The predictions of miRNA-target interactions and the information about the miRNA family members with their seed (*i.e*. positions 2 to 8 at the 5′-end of the mature miRNA sequence) were downloaded from TargetScan web site Release 7.0 (August 2015)^61^. The experimentally validated miRNA-target interactions were downloaded from miRTarBase web site Release 6.1 (September 2015)^62^.

Given a genes list, for each selected miRNA, the hypergeometric test was used to calculate the significance (p-value <0.05) of the enrichment in targets of that miRNA. The p-value is computed as

1 $$p=1-\sum _{i=0}^{X-1}\frac{(\begin{array}{c}K\\ i\end{array})(\begin{array}{c}M-K\\ N-i\end{array})}{(\begin{array}{c}M\\ N\end{array})}$$

where *M* is the number of all transcripts predicted (validated) as miRNA targets in TargetScan (miRTarBase), *K* is the number of all targets of the selected miRNA, *N* denotes the size of the input genes list included in TargetScan (miRTarBase), and *X* is the number of transcripts of the input gene list predicted (validated) as target of that miRNA.

### Functional and motifs enrichment analysis

The associations between selected switch genes and functional annotations such as KEGG pathways^85^ and GO terms^86^ were analyzed by using FIDEA^39^ and GSEA^40^ web tools. Binding motif enrichment analysis in promoter regions (identified as genomic regions spanning from −450 to +50 nucleotides with respect to transcription start sites) was performed by Pscan^47^, which employs the JASPAR 2018 motif collection^87^. A p-value < 0.05, after adjustment for multiple testing performed with the Benjamini-Hochberg method, was set as threshold to identify functional annotations and regulatory motifs significantly enriched amongst the selected gene lists.

### Kaplan-Meier

In order to evaluate the clinical relevance of each gene identified by SWIM as involved in the shift from TPCs to DGCs, we performed Kaplan-Meier analyses^88^ by using clinical and expression data provided by The Cancer Genome Atlas (TCGA) portal^50,51^, relating to 161 patients affected by glioblastoma. The patient samples are split into two groups (called low-expression and high-expression) according to the expression level of a given switch gene with respect to distribution of the expression values of all switch genes. In particular, low- and high-expression groups refer to patients with expression levels lower than or greater than the 50^*th*^ percentile, respectively. For each patient cohort, the cumulative survival rates are computed for each switch gene according to the Kaplan-Meier method^88^. A log-rank test was performed to evaluate the p-value associated with each switch gene: the lower the p-value, the better the separation between the prognoses of the two groups. Finally, the switch genes were sorted by increasing p-values in order to identify those that are best at distinguishing the two patient groups.

## Electronic supplementary material

Supplementary Figures

Supplementary Table S1

Supplementary Table S2

Supplementary Table S3

Supplementary Table S4

Supplementary Table S5

Supplementary Table S6

Supplementary Table S7

Supplementary Table S8

Supplementary Table S9

Supplementary Table S10

Supplementary Table S11
