# Publisher Correction: Computational identification of specific genes for glioblastoma stem-like cells identity

**DOI:** 10.1038/s41598-018-28287-z

**Published:** 2018-07-09

**Authors:** Giulia Fiscon, Federica Conte, Valerio Licursi, Sergio Nasi, Paola Paci

**Affiliations:** 1 Institute for Systems Analysis and Computer Science “Antonio Ruberti”, National Research Council, Rome, Italy; 2 SysBio Centre of Systems Biology, Rome, Italy; 3 Department of Biology and Biotecnology - Charles Darwin, “Sapienza” University of Rome, Rome, Italy; 4 Institute of Molecular Biology and Pathology (IBPM), National Research Council (CNR), Rome, Italy

Correction to: *Scientific Reports* 10.1038/s41598-018-26081-5, published online 17 May 2018

Eleven supplementary tables were omitted from the original version of this Article. This has been corrected in the HTML version of the Article; the PDF version was correct at time of publication.

