# Supplementary material for: Computational identification of specific genes for glioblastoma stem-like cells identity
Source: Sci Rep. 2018 May 17;8:7769. doi: 10.1038/s41598-018-26081-5 (PMC5958093; doi:10.1038/s41598-018-26081-5)
Supplement: Supplementary file 1 — Supplementary Figures [file 41598_2018_26081_MOESM1_ESM.pdf]

# Computational identification of specific genes for glioblastoma stem-like cells identity

**Giulia Fiscon**[1,2,†], **Federica Conte**[1,2,†], **Valerio Licursi**[1], **Sergio Nasi**[3,4,◇], **and Paola Paci**[1,2,*,◇]

[1]Institute for Systems Analysis and Computer Science “Antonio Ruberti”, National Research Council, Rome, Italy
[2]SysBio Centre of Systems Biology, Italy
[3]Department of Biology and Biotecnology - Charles Darwin, “Sapienza” University of Rome, Italy
[4]Institute of Molecular Biology and Pathology (IBPM), National Research Council (CNR), Italy
[*]corresponding author: paola.paci@iasi.cnr.it
[†]equal contributors
[◇]co-senior authors

## ABSTRACT

Glioblastoma, the most malignant brain cancer, contains self-renewing, stem-like cells that sustain tumor growth and therapeutic resistance. Identifying genes promoting stem-like cell differentiation might unveil targets for novel treatments. To detect them, here we apply SWIM – a software able to unveil genes (named switch genes) involved in drastic changes of cell phenotype – to public datasets of gene expression profiles from human glioblastoma cells. By analyzing matched pairs of stem-like and differentiated glioblastoma cells, SWIM identified 336 switch genes, potentially involved in the transition from stem-like to differentiated state. A subset of them was significantly related to focal adhesion and extracellular matrix and strongly down-regulated in stem-like cells, suggesting that they may promote differentiation and restrain tumor growth. Their expression in differentiated cells strongly correlated with the down-regulation of transcription factors like OLIG2, POU3F2, SALL2, SOX2, capable of reprogramming differentiated glioblastoma cells into stem-like cells. These findings were corroborated by the analysis of expression profiles from glioblastoma stem-like cell lines, the corresponding primary tumors, and conventional glioma cell lines. Switch genes represent a distinguishing feature of stem-like cells and we are persuaded that they may reveal novel potential therapeutic targets worthy of further investigation.

## Supplementary Figures

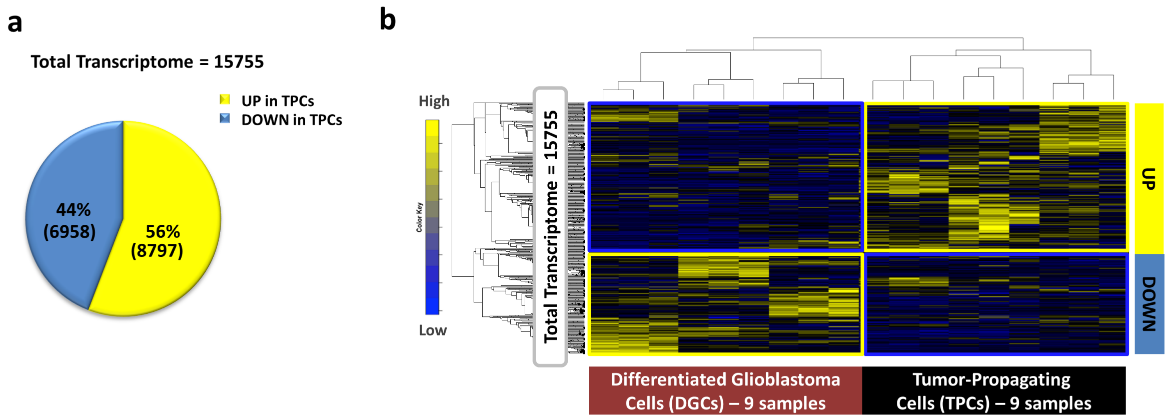


**Figure 1. Global gene expression analysis of tumor promoting and differentiated glioblastoma cells.** (**a**) Percentages of genes that were up-regulated and down-regulated in the tumor-propagating cells (TPCs) in comparison to differentiated glioblastoma cells (DGCs). (**b**) Dendrogram and heat map of the comprehensive human transcriptome in glioblastoma cells. The expression profiles of the differentially expressed genes are clustered according to genes (rows) and cells (columns) in the glioblastoma data matrix by using Pearson correlation distance as metrics. Heat map colors represent different expression levels increasing from blue to yellow.

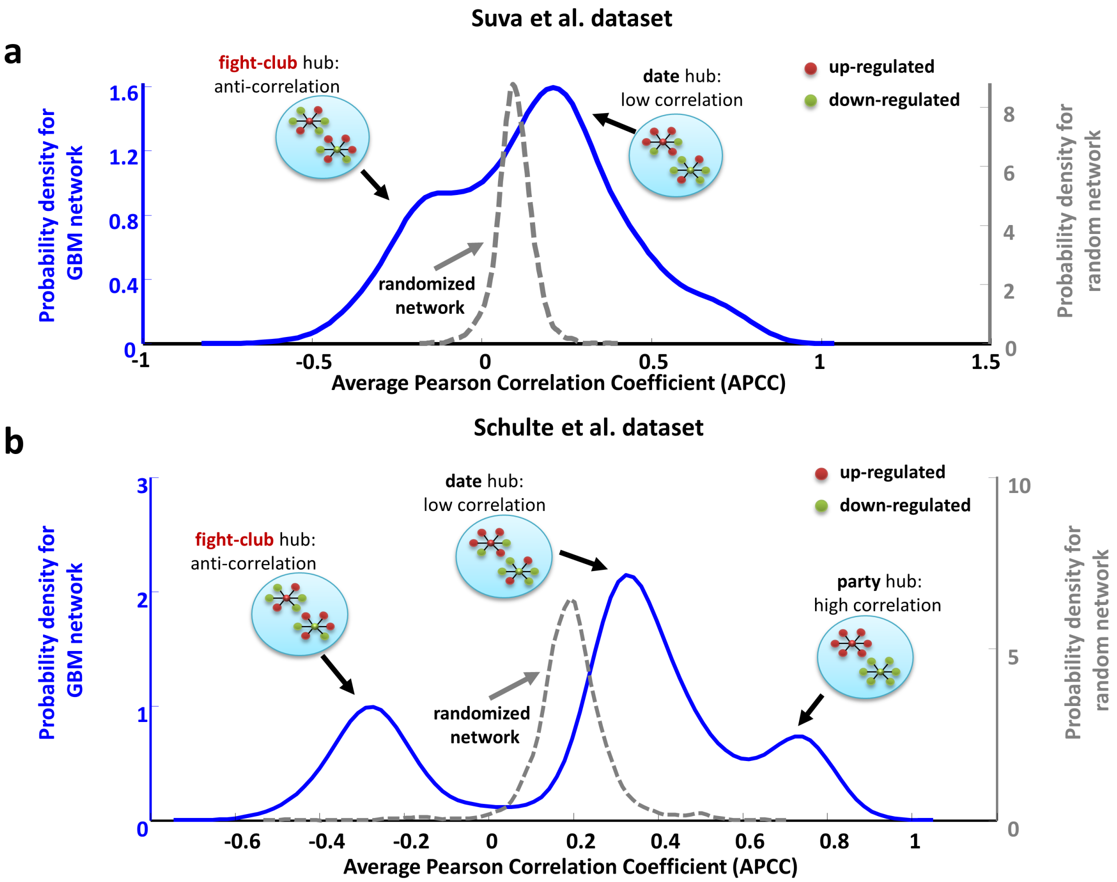


**Figure 2. APCC distribution for the glioblastoma correlation network for Suva et al. dataset (a) and for Schulte et al. dataset (b).** (**a**)-(**b**) Probability distributions of APCC for hubs identified in the correlation network built from the glioblastoma expression dataset (blue solid lines) of Suva et al. (**a**) and of Schulte et al. dataset (**b**) and their randomized counterparts obtained by shuffling the edges but preserving the degree of each node (grey dashed lines). Differing from the randomized case, the true APCC distributions show a trimodal pattern where peaks correspond to party hubs (high positive APCC), date hubs (low positive APCC), and the fight-club hubs (negative APCC).

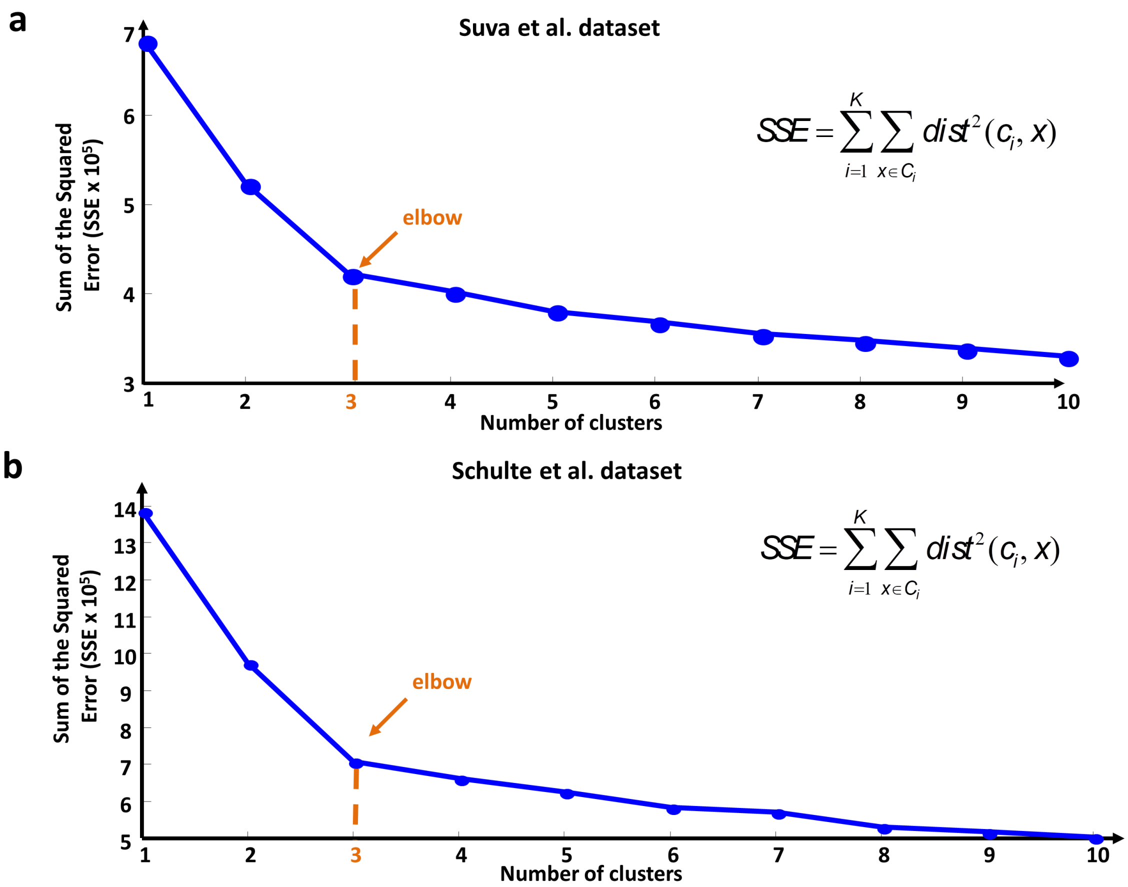


**Figure 3. Scree plot for Suva et al. dataset (a) and for Schulte et al. dataset (b).** (**a**)-(**b**) The x-axis represents the number of clusters, while the y-axis represents the Sum of the Squared Error (SSE). The SSE is computed as the sum of the distance of each object to its closest centroid. As a distance measure, SWIM makes use of $dist(x,y) = 1 - \rho(x,y)$, where $\rho(x,y)$ is the Pearson correlation coefficient between the expression profiles of nodes $x$ and $y$. Finally, a reasonable number of clusters is chosen on the basis of the elbow position.

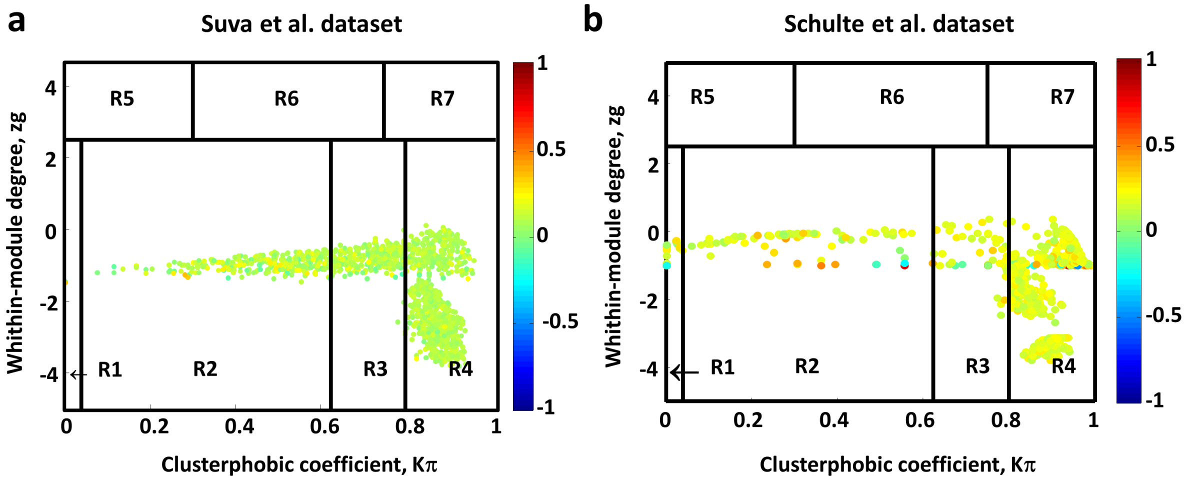


**Figure 4. Heat cartography map of nodes of random network for Suva et al. dataset (a) and for Schulte et al. dataset (b).** (**a**)-(**b**) Dots correspond to nodes in the random correlation network of Suva et al. dataset (**a**) and of Schulte et al. dataset (**b**). Nodes are distributed across seven regions (R1 to R7) according to their clusterphobic coefficient $K_\pi$ (x-axis), which is a measure of the "fear" of each node of being confined in its own cluster, and according to their within-module degree $z_g$ (y-axis). Each node is colored according to its APCC value.

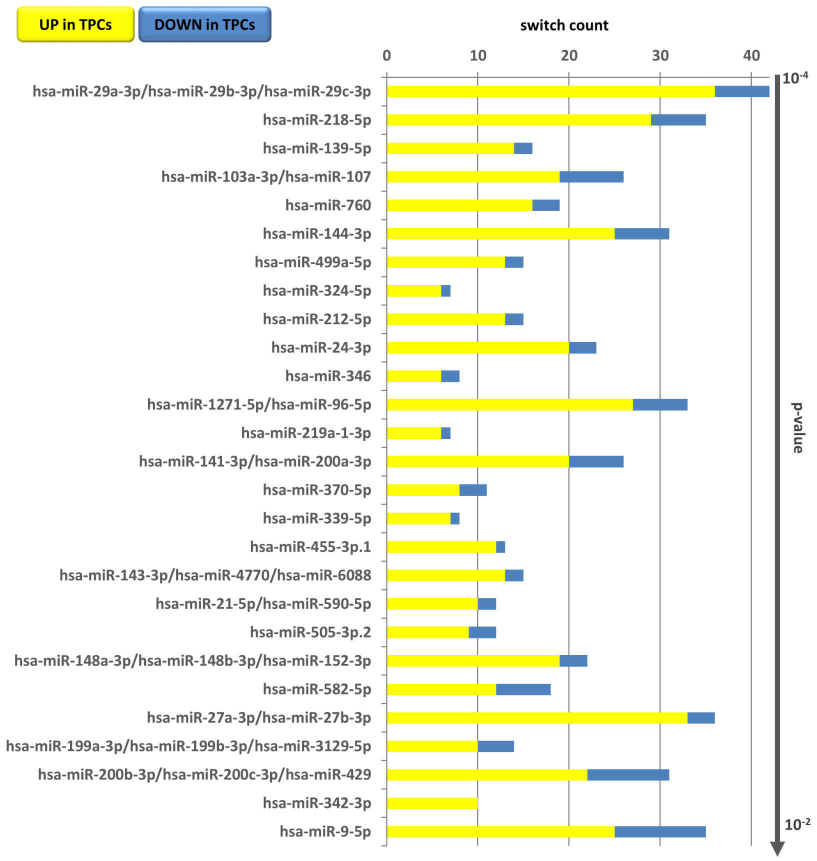


**Figure 5. miRNAs enrichment in the 3'UTR of switch genes.** The stacked bar plot represents all miRNA family members found to be significantly enriched (p-value $< 0.05$) in the lists of switch genes identified by SWIM in GBM dataset of Suva et al. together with the count of their targets. The miRNAs are sorted according to the increasing p-values. Yellow and blue bars refer to up- and down-regulated switch genes in the DGCs, respectively. miRNA-target interactions are based on TargetScan predictions (http://www.targetscan.org/vert_71/)
.
